# A multi-stage approach to support timely health policy decisions during crisis: the *fast-track Delphi*

**DOI:** 10.1186/s12889-024-20903-0

**Published:** 2024-12-18

**Authors:** Camille Velarde Crézé, Olivier Duperrex, Luc Lebon, Vincent Faivre, Myriam Pasche, Jacques Cornuz

**Affiliations:** 1 Department of Health Promotion and Prevention, University Center for Primary Care and Public Health (Unisanté), University of Lausanne, Route de Berne 113, Lausanne, CH – 1010 Switzerland; 2 Direction of Finances – Informatics Systems and Digital Transformation Unit, University Center for Primary Care and Public Health (Unisanté), University of Lausanne, Lausanne, Switzerland; 3 University Center for Primary Care and Public Health (Unisanté), University of Lausanne, Lausanne, Switzerland

**Keywords:** Delphi, Expert opinion, Crisis, Rapidity, Policymaking support, Consensus, Consensual agreement, Nominal group technique, Toolkit, Methodological development

## Abstract

**Background:**

Scientists can play an important role in policymaking by providing evidence and consensual expert opinion on the state of scientific knowledge. Delphi surveys have been widely used to develop consensus on a topical issue, yet not compatible with public health crisis situations requiring rapid decisions. We developed a *fast-track Delphi* process, providing experts with a structured approach to rapidly develop and quantify consensus in support of informed policy decisions.

**Methods:**

We identified key elements of consensus-building techniques through a literature review and derived methodological procedures that served as the basis for the elaboration of the new process. Selected methodological experts provided advice on necessary adjustments. The process was pilot tested using a real-world public health issue.

**Results:**

The *fast-track Delphi* process is a hybrid approach between a conventional Delphi and the nominal group technique: one group session followed by two rounds of e-questionnaire, with predefined steps. We developed an ad hoc toolkit (REDCap templates, R code for analysis and production of reports, user guide) to overcome time constraints, which we pilot tested with experts. The feasibility test conducted in 18 days in the field of tobacco control demonstrated the applicability and usefulness of the process in real-world conditions.

**Conclusions:**

We strongly believe that this *fast-track Delphi* process has the potential to help inform policy decisions in various types of crises, including emerging diseases or novel potentially harmful products.

**Supplementary Information:**

The online version contains supplementary material available at 10.1186/s12889-024-20903-0.

## Introduction

Scientists may endorse an important role in policy decisions. Although they do not make policy decisions per se, they contribute by advancing knowledge and informing on the implications of findings, based on their expertise [1]. In times of public health crisis, national and regional COVID-19 task forces are examples of the role scientists played during the pandemics in informing policymakers of the current state of scientific opinion and knowledge, yet also highlighting challenges for a sound and efficient collaboration between scientist and policy makers [2].

One way of improving science impact in policies when limited evidence or overwhelming, conflicting information is available, is for experts to develop consensus in their field as much as possible, to help policymakers make informed choices. A Delphi survey (further referred to as conventional Delphi) is one widely used and proven method for developing and quantifying consensus among experts on a topical issue [3–6]. Experts do not meet but answer repeated questionnaires (often two or three rounds) so that their opinions and comments can be refined in light of the group responses to reach a final consensus [7, 8].

Scientific uncertainty, lack of data, a rapidly changing context, and short timelines for policy decisions characterize public health crises such as we experienced in the COVID-19 pandemics. Therein, the conventional Delphi approach is not suited to these time constraints, as this approach often takes months, if not years, to complete the entire process [3]. Yet, scientists (e.g., a task force, a non-decisional scientific council) should be methodologically equipped to provide consensual opinions to policymakers within a limited timeframe. Therefore, we sought to develop and test a generic *fast-track Delphi* process and its associated technical tools aiming at helping scientists to develop and quantify consensus in a crisis context, to support the most rapid and informed policy decision-making.

## Materials and methods

**Fig. 1:**
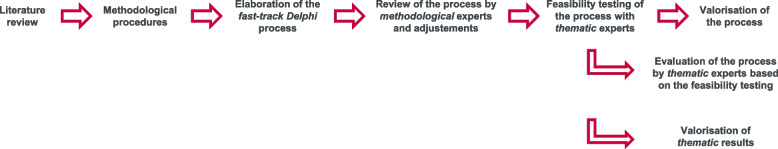
Strategy used to develop and test the fast-track Delphi process

Figure 1 illustrates the strategy we used to develop and test the *fast-track Delphi* process, with each development phase described in more details in the following sections. The *fast-track Delphi* process itself, which is the product of our methodological development, is described in detail in the “Results” section below.

### Literature search strategy and elaboration of methodological procedures

We conducted a narrative literature review [9] to identify key elements of the conventional Delphi and other consensus-building techniques. To complete the available references known to our group, we first searched Medline/PubMed up to March 2022 using a combination of the keywords “Delphi”, “guidelines”, “good practice”, “consensus development”, “consensus technique”, “modified Delphi”, and limited our selection to books and documents, meta-analyses, and (systematic) reviews. We excluded individual Delphi surveys or other original research studies using consensus development techniques at this stage. We then reviewed the references of the papers identified so far to include other methodological reviews of the conventional Delphi and other related techniques. We then used this literature review as a basis to identify methodological procedures, focusing on: Participants, Questions & Round 1, Rounds 2 & followings, Data analysis & feedback, Definition of consensus, Interpretation & reporting, Project management.

### Development and review of the *fast-track Delphi* process, and its associated toolkit

We then developed the *fast-track Delphi* process combining key points of the conventional Delphi and other related consensus-building techniques. We then formalized and described each step, from selecting and recruiting experts to producing a synthesis of results for policymakers. Finally, we listed all steps requiring technical assistance and identified an appropriate tool (e.g., software) for developing an ad hoc technical process, and for which internal resources were available.

Selected experts in conventional Delphi surveys and/or qualitative research in various fields from our institution and outside of it (hereafter *methodological experts*) reviewed the methodological procedures along with the newly developed *fast-track Delphi* process and provided advice on adjustments to improve the process.

### Pilot phase: feasibility testing of the *fast-track Delphi* process in a real setting

We conducted a pilot to test the practicality of the *fast-track Delphi* process. We chose the regulation of new disposable electronic cigarettes (*puffs*) because of scientific uncertainty, lack of data, and public health concerns on that topic. We conducted this pilot over 18 days, from June 17 to July 4, 2022, by selecting and recruiting experts in the field of tobacco control (hereafter *thematic experts*) and going through all steps of the process described in the results and using the technical tools we developed. The thematic results of the pilot belong to the field of tobacco control and are available elsewhere (in French) [10]. We report here methodological results that demonstrate the feasibility and limitations of the *fast-track Delphi* process. We used proportions for categorical variables and summary of distribution for continuous ones (median, min, max, interquartile range).

In addition, we obtained feedback on the process from these thematic experts through an e-questionnaire (categorical, continuous visual analogue scale and open-ended questions) sent on July 15, 2022, with a simple analysis (frequencies for categorical variables, median and interquartile for continuous variables). Based on their feedback and self-critique, we adjusted the *fast-track Delphi* process by adding some extra methodological procedures.

## Results

### Methodological procedures

We started with some references previously identified by our group from one’s own experience with conventional Delphi surveys [5, 11–13], selected five references from our PubMed search [8, 14–17], and eight others from relevant hit references [3, 4, 6, 7, 18–21]. This literature review provided us with information on the availability and methodological details of four consensus-building methods, namely the conventional Delphi survey (as well as various versions of so-called *modified* conventional Delphi surveys), the Nominal Group Technique (NGT), the RAND/UCLA Appropriateness Model (RAM), and the consensus development conference. We then formulated methodological procedures based on this review (Supplementary Table S1). Seven methodological experts out of the 8 invited (87.5%) reviewed an earlier version of Table S1 (available on request).

### Steps of the *fast-track Delphi* process

Based on the methodological procedures and methodological experts’ advice, we developed the *fast-track Delphi* process, which allows for the development and quantification of consensual agreements between thematic experts within two to three weeks. This approach is a hybrid process between a conventional Delphi and a modified version of the NGT [22].

**Fig. 2:**
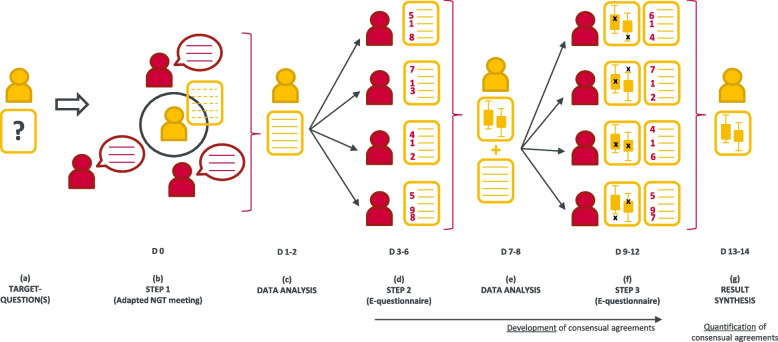
Overview of the *fast-track Delphi* process. Members and productions from the organizational team are shown in yellow, while thematic experts and their productions are shown in red. The process calendar is indicated with days (D) number. **a** Target question(s) are formulated by the organizational team and sent to experts upstream from step 1. **b** Step 1 consists in addressing this(ese) target question(s) with the panel of experts during a meeting organized following an adapted Nominal Group Technique (NGT) procedure, to generate and prioritise a list of thematic proposals. **c** The organizational team analyzes these proposals and edits them into statements to form step 2 e-questionnaire. **d** Step 2 consists in distributing this e-questionnaire to the panel of experts, who express their opinion on the statements. **e** The organizational team analyzes data (experts’ opinion) from step 2 and produces one group report and several individualized reports. Statements are refined based on responses and comments from step 2 to form a new e-questionnaire. **f** Step 3 consists in distributing this e-questionnaire to the panel of experts, who re-express their opinion considering group responses and their individual responses from step 2. **g** The organizational team analyzes data (experts’ opinion) from step 3 and produces a result synthesis. Steps 2 and 3 enable the development of consensual agreements, while the synthesis enables the quantification of final consensual agreements

Figure 2 provides an overview of a *fast-track Delphi* process and Table 1 an overview of the human resources and associated skills required in the organizational team to conduct such process in a timely manner [12–14].

**Table 1:** Human resources and corresponding skills needed to conduct a *fast-track Delphi*

Quantity^a^	Role	Tasks	Skills	Involvement
1	Project coordinator	Piloting the project, coordinating the various actors, ensuring compliance with the timeline	Project management	Beforehand and covering the whole process
1–2	Thematic experts	Formulating target question(s), providing (and summarizing) relevant pieces of literature, identifying thematic expert participants, formulating statements	Detailed and up-to-date knowledge of the thematic field	Beforehand and data analysis between steps
1	Administrative support	Scheduling expert recruitment contacts, launching e-questionnaire steps and sending reminders, ensuring information communication, ensuring step 1 meeting logistics	Administrative skills	Covering the whole process
2	Step 1 meeting moderators	Step 1 meeting animation: moderation (one person) and real-time projected notetaking (one person)	Group animation skills, e.g., allotted speaking time management, listening and synthetizing skills, real-time notetaking	Beforehand and Step 1 meeting
1	Data analyst	Data analysis, production of e-questionnaires, production of group results and individualized feedbacks	Knowledge of data analysis, descriptive statistics and visual/graphical result display, synthesizing skills, skills in the management of technical tools used	Covering the whole process
1–2	Technical experts	Development of an electronic platform hosting e-questionnaires and a process ensuring data processing and reporting	Informatics and coding skills, descriptive statistics, visual and graphical displays	Beforehand and on consultation during the whole process

^a^One person can endorse multiple roles as a function of his/her skills

**Fig. 3:**
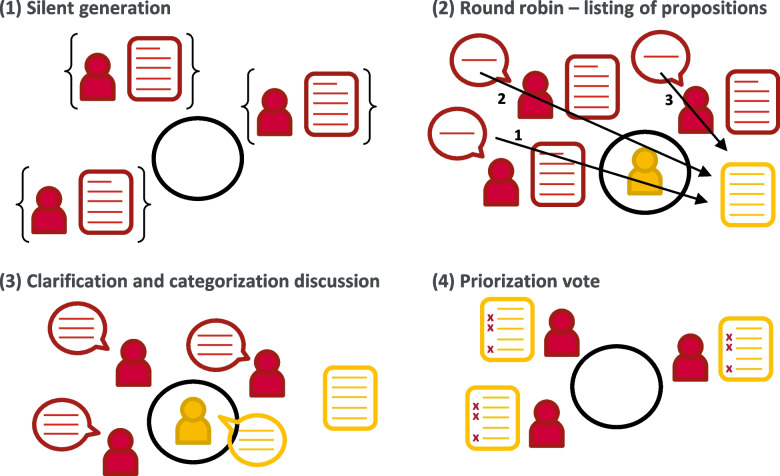
Illustration of the four phases of an adapted version of the Nominal Group Technique (NGT; step 1 of the *fast-track Delphi* process). These four phases take place during a 1h 30 to 2h-meeting. Members and productions from the organizational team are shown in yellow, while thematic experts and their productions are shown in red

First, the organizational team identifies and recruits at least 20 to 25 thematic experts from an existing task force, local or national professional groups, or from the authorship of key publications. The rationale for choosing these target numbers is based on a predicted 30% refusal at recruitment and dropout rate throughout the process, which ensures a final sample of 15 participants [3, 12, 14, 19, 21, 23]. The team conducts recruitment on a one-to-one basis as much as possible, and the goal of consensus development is explained to experts. Based on demand from policymakers, civil society, and/or scientists themselves, the organizational team conducts a rapid review to formulate one (or at most two) target question(s) to address the issue (Fig. 2, panel (a)), and provide relevant papers to the panel. This initial rapid review on a focused question might need to be broadened if insufficient evidence is available. Then, the process itself consists of three main steps:

- Step 1: an adapted Nominal Group Technique (NGT) – Fig. 2, panel (b) – Experts meet and brainstorm as a group to provide topical proposals in response to the target question(s), and select the most priority ones to address subsequently [14, 22]. The four phases of the adapted NGT are illustrated in Fig. 3
- Step 2: e-questionnaire – Fig. 2, panels (c-e) – Experts express their opinion on the topical proposals raised and selected in step 1
- Step 3: e-questionnaire and result synthesis – Fig. 2, panels (f-g) – Using a second e-questionnaire and in light of step 2 results, experts express again their opinion on the statements that have not yet reached consensual agreement, and reformulated according to results and comments provided in step 2 [5].

More technical details on these three steps are available in the Supplemental material.

#### Types of questions and definitions of agreement and consensus

We defined three types of questions to be included in e-questionnaires (steps 2 and 3):

- type 1 question – expression of the level of agreement with the statement via a *rating* on a scale of 1 (= total disagreement) to 9 (= total agreement)
- type 2 question – single response among several possible options
- type 3 question – multiple response (maximum three choices) among several possible options.

Type 1 questions are central to Delphi surveys, in that they enable experts to take position on affirmative statements [12, 23]. Therefore, the last step of a *fast-track Delphi* process (i.e., e-questionnaire of step 3) should only contain type 1 questions. Type 2 and 3 questions, on the other hand, might be included in the e-questionnaire of step 2 with the aim to clarify experts’ topical propositions and orient future statements.

The definition of consensual agreement we propose for a *fast-track Delphi* process is based on type 1 questions (affirmative statements). Most responses are likely to have a skewed or even polarized distribution. In accordance with the methodological procedures, we thus defined two data components [4, 5, 13]. For each statement, the center of the data (median) represents the level of experts’ *agreement.* We split the scale in three equal intervals and chose a threshold of 7 or more out of 9 to consider that an agreement with the statement was reached (3 or less out of 9 for a disagreement with the statement). The dispersion of responses around the center of the data (interquartile interval, IQR) represents the *consensus*. We chose a threshold of IQR ≤ 3 points of the scale to consider that experts reached a consensus. In the *fast-track Delphi* process, we aim at reaching *consensual agreements*, that is, a group response for a given statement with a median of 7 (or more) and an IQR of 3 (or less) points of the scale [8, 13]. A consensual *dis*agreement would have a median of 3 (or less) and an IQR of 3 (or less) points of the scale. When considering response options for type 2 and 3 questions, we propose considering that experts reached a consensus when the response option is chosen by at least two thirds of the experts (≥ 66%).

### Technical toolkit development

We identified three key elements that required technical support:

- To facilitate the meeting in step 1, we used a mind map management software projected with a beamer during the brainstorm phase (not anonymous) and an online application to allow easy and anonymous voting for the final phase of step 1 (prioritisation) [4, 14]
- To create and administer the e-questionnaires in steps 2 and 3, we used an electronic survey management platform that guaranties confidentiality and accountability such as REDCap [24, 25]
- To analyze the data and to produce generic reports (descriptive statistics and visual description of responses) and individualized reports (each expert’s own response against the group response distribution), we created a tool coded in R language, using the RStudio interface [26, 27].

The R code, the associated user guide, the templates for question types to import in REDCap and a demonstration code and anonymized dataset will be made available upon request to the corresponding author. More details on the development of this toolkit are also described in the Supplemental material. We strongly suggest that the organizational team be familiar with the tools and techniques used in every step ahead of conducting a *fast-track Delphi* process, as this will be critical for a smooth process running.

### Pilot: feasibility testing and evaluation of the *fast-track Delphi* process

**Fig. 4:**
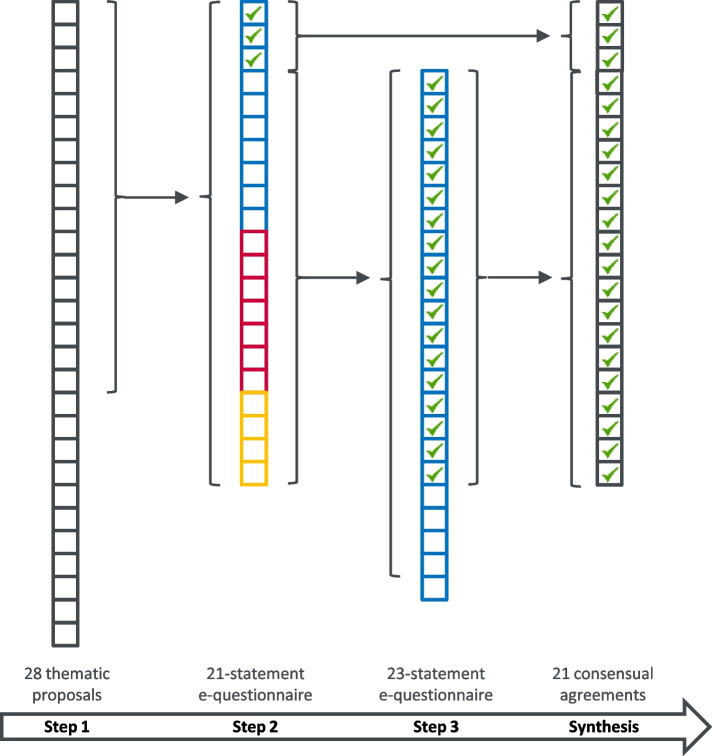
Pilot (feasibility test) – Flow of statements throughout the three steps of the *fast-track Delphi* process. A square represents a thematic proposal (step 1) or question/statement (steps 2 and 3). For steps 2 and 3, the type of question is symbolized by the color of the box (blue = type 1; red = type 2; yellow = type 3). For type 1 statements, the green ticks represent the consensual agreements reached

We tested the process and the technical toolkit by conducting a *fast-track Delphi* on the regulation of disposable electronic cigarettes in French-speaking Switzerland (target question: *“On which aspects should puff-like disposable electronic cigarettes be regulated, and how?”)*. This feasibility test lasted 18 calendar days (day 1: step 1 (NGT meeting); day 18: production of the executive summary presenting final results and key messages), and included 23 experts (61% of invited experts, acknowledging an invitation sending 31 calendar days before day 1 of the process). Figure 4 shows the flow of statements throughout steps 1 to 3 of the *fast-track Delphi* feasibility test. Twenty-one statements out of 26 (80%) reached consensual agreement at the end of the process, covering regulation aspects pertaining to the composition of puff-like disposable electronic cigarettes, product marketing, sales and consumption restrictions as well as the implementation of control measures. More details on the methodological results of this feasibility test are available in the Supplemental material.

An evaluation questionnaire was sent to all 23 respondents of step 2. Results are shown in Table 2 and more details are available in the Supplemental material. We then added a few extra methodological procedures to Table S1 (marked with an *), based on thematic experts’ feedback and lessons learnt from this pilot.

**Table 2:** Results from the evaluation e-questionnaire

Question	Type of question *(Answer options)*	N (% of respondents)^a^	Result
**Reference documents**			
Relevance and usefulness of reference documents upstream from step 1	Unique choice question *(Yes, No, No answer)*	*N* = 16 (100%)	Yes: *n* = 16 (100%)
**Step 1 (adapted NGT)**			
Added value of an adapted NGT for step 1	Visual analog scale *(0-no added value, to 10-very high added value)*	*N* = 16 (100%)	Med ± IQR: 8.5 ± 1.3 points
Added value of the presential modality for the adapted NGT (step 1)	Visual analog scale *(0-no added value, to 10-very high added value)*	*N* = 9 (100% of step 1 participant respondents)	Med ± IQR: 9.0 ± 2.0 points
Quality of step 1 meeting moderation during the feasibility test	Visual analog scale *(0-very inadequate, to 10-very adequate)*	*N* = 9 (100% of step 1 participant respondents)	Med ± IQR: 9.0 ± 2.0 points
**Step 2 (e-questionnaire)**			
Adequacy of step 2 e-questionnaire content with regards to step 1 results	Visual analog scale *(0-step 1 results not at all taken into account, to 10-step 1 results fully taken into account)*	*N* = 9 (100% of step 1 participant respondents)	Med ± IQR: 9.0 ± 2.0 points
**Step 3 (e-questionnaire)**			
Adequacy of step 3 e-questionnaire content with regards to step 2 results	Visual analog scale *(0-step 2 results not at all taken into account, to 10-step 2 results fully taken into account)*	*N* = 16 (100%)	Med ± IQR: 9.0 ± 1.0 points
Use of the individualized step 2 results report when completing step 3 e-questionnaire	Unique choice question *(Yes, No, No answer)*	*N* = 16 (100%)	Yes: *n* = 16 (100%)
**General**			
Format adequacy (ease of use) of e-questionnaires	Visual analog scale *(0-very inadequate, to 10-very adequate)*	*N* = 16 (100%)	Med ± IQR: 9.0 ± 1.0 points

*Med* median, *IQR* interquartile range

^a^E-questionnaire distributed to the 23 thematic experts having participated in the pilot phase of the *fast-track* Delphi process

## Discussion

Our study aimed at developing and testing a process allowing consensus building and quantification among experts to use in the context of a public health crisis. This process should be 1) adapted to a public health crisis, i.e., particularly in terms of speed required to support policy makers in a timely manner, and 2) generic, to allow consideration of any relevant issue, no matter the thematic, at the required time. We provide here details on a new structured *fast-track Delphi* approach with its associated tools. We also report the results of a real-world feasibility test using the technical tools developed to implement the process, as well as positive feedback from participating experts.

As far as we know, such an adapted process that enables consensus building in public health crisis situations does not exist, or at least is not formalized as such in the literature [7, 12, 13]. The robustness of the conventional Delphi approach lies in its multiple rounds, anonymous process with feedback. The structure in multiple rounds, itself facilitated by the anonymity of the process – referred to as the principle of ‘quasi-anonymity’, i.e., the identity of the experts is known to the organizational team and vice versa, but their opinion within the process remains anonymous – allows the gradual development of consensus [7, 8, 18, 20]. Giving feedback to experts in the form of a statistic and visual description of responses favors consensus building in light of the group’s responses [5, 14]. The NGT, on the other hand, is considered an extremely time-efficient technique, i.e., a compromise between standardized (less prone to bias) methodology due to the well-established group moderation protocol and the time needed to complete the process [14, 15, 19]. It allows for rapid generation of experts’ opinion yet does not provide a context in which consensus can be built based on the progressive integration of the group’s opinion.

We developed a hybrid procedure that combines key aspects of the conventional Delphi and the NGT, to find a compromise between methodological rigor and the need for speed. Some studies combining aspects of both approaches can be found in the literature [6, 14, 28]. However, they do not use an established combination of both methods, nor are they conducted in a very limited time frame, which is a challenge but an essential feature in times of crisis. The originality of our work lies in 1) formalizing a hybrid process between the two proven techniques with detailed descriptions of each step, and 2) the development of an associated toolkit that allows overcoming technical obstacles to complete the process in two to three weeks.

Our study and the process we developed have several limitations. First, the (adapted) NGT for step 1 cannot preserve the anonymity of the experts when expressing ideas. In a conventional Delphi approach, the principle of quasi-anonymity can only be respected by using (e-)questionnaire rounds without experts brainstorming as a group [12, 23, 29]. The desirability bias, i.e. the conscious or unconscious will to report desirable attributes and opinions, may be even greater in a meeting of experts than in an online survey [3, 5, 19, 20]. In order to make the best use of group dynamics in step 1 without distorting the outcome (e.g. when more vocal or renowned experts dominate the discussion), it is crucial to carefully select the moderator(s) of the adapted NGT who have good group animation skills [4, 5].

Second, we would like to emphasize how important, but also difficult, it is for the organizational team to remain neutral when formulating statements. This challenge arises not only in the *fast-track Delphi* process, but also in a conventional Delphi [3]. The team members who create the e-questionnaires are subject matter experts themselves (i.e., necessary to achieve accurate and relevant statement formulation), yet they should not express their opinions or take a stand when formulating statements. The process aims to collect, analyze, and regroup the opinions of the participating experts, not those of the organizational team [12]. To this end, we suggest a double check of e-questionnaires before they are sent to the participating experts, i.e., from both thematic and formulative points of view. At least one reviewer should not be involved in running the process or a participant. Although this is not error-free, it ensures high quality of the e-questionnaires through a multi-expert view, e.g., by avoiding organizers unintentionally stating their opinion or formulating double-item statements [20].

Third, the *fast-track Delphi* process itself would benefit from two complementary structures or processes. A pre-selection and recruitment process to assemble the expert panel in advance of a crisis would allow for better responsiveness when a specific issue arises that requires rapid development and quantification of expert consensus [30]. In addition, a structured process with appropriate resources would allow to monitor and gather scientific knowledge ahead of step 1. Even if the panelists recruited for the *fast-track Delphi* process are experts on the topic, they might have specific expertise on a particular aspect of the issue. A rapid literature review would provide information for the expert panel, as appropriate, to begin the process on a common reference basis [3, 6].

Fourth, testing the feasibility is not the same as validating the *fast-track Delphi* process as a new methodology [12]. Repeating the test with the same target question and a different panel could serve to validate the results (context- and knowledge-dependent at time T), not necessarily to validate the method [4, 7, 20]. However, we are confident that the process is robust, as it evolved from two proven approaches, has been reviewed by methodological experts and adapted following their advice [8].

Finally, we would like to point out the interpretive limitation of such procedures. One should not over interpret the results of a *fast-track Delphi* survey, since reaching a consensus among experts does not mean that new scientific evidence has been established [5, 6, 12, 20]. One might also keep in mind that the chosen definition and thresholds for consensual agreement remain pragmatic, i.e., as no consensus exist in the literature on their specific definition. These limitations are not specific to the newly developed *fast-track Delphi* process, but inherent to any consensus development study: consensus development is a process that can help lay the groundwork for policy decisions, not a scientific method for creating new knowledge [4]. Moreover, results should always be contextualized to consider the state of knowledge at the time when experts express their opinion. We advise conducting the process with rigor when there is a need to support important decisions, while remaining open to revision as new knowledge emerges [31]. If judged necessary to improve the clarity of key messages addressed to policymakers – or if asked by policymakers themselves –, one might add after step 3 a short supplementary step (24 to 48 h) consisting in a second prioritisation vote – experts using again a similar online technical tool as used by the end of step 1 to select the top-3 or top-5 statements, among those having reached consensual agreement, that they consider priority to address by policy decisions [1].

Our study has several strengths. First, we tested the newly developed *fast-track Delphi* process on a public health problem, in a context that has several aspects of a crisis, such as lack of scientific data and concerns of several public health stakeholders, uncertainty, and rapidity of spread of the phenomenon. This real-world feasibility test demonstrated the ability of the process to develop and quantify consensual agreements for a large portion of the statements generated and examined throughout the three steps of the process, in less than three weeks. The process also benefited from feedback and adjustments from participating experts. Second, we developed and tested a technical toolbox to ensure a timely completion of the process. Third, we want to emphasize the positive feedback of thematic experts on the adapted NGT in step 1. This might have increased loyalty to the group and the goal of the process, a result that is also reflected in the response rates we were able to achieve [13, 18]. Finally, while this process was developed for a public health crisis context, it might also show advantages in other situations, e.g., by enhancing the scientific knowledge context coherence in a limited time period.

Although our study aimed to obtain consensus from a panel of scientific experts, it could be used with panels of a variety of stakeholders, including patients, beneficiaries, politicians, citizens, either in a mixed group to reach a consensus or as separate groups to identify differences between stakeholders [32].

## Conclusion

We developed and tested a new *fast-track Delphi* process, which consists of a structured combination of key aspects of a conventional Delphi and an adapted NGT, to support policymakers make rapid (two to three weeks) and informed decisions in public health crises. We clearly identified and detailed each step of the process and developed a toolbox that allows to deliver expert consensus to policy makers in a timely manner. The feasibility test conducted on a public health issue in the field of tobacco control demonstrated the applicability and usefulness of the process in a real-word condition. We strongly believe that this *fast-track Delphi* process has the potential to help inform policy decisions in various types of crises, including emerging diseases or novel potentially harmful products.

## Supplementary Information

Supplementary Material 1.

Supplementary Material 2.

## Data Availability

Data collected during the pilot phase will be made available as a demonstration dataset (anonymized; *.RData format; DOI: 10.16909/dataset/41), together with the code itself (DOI: 10.16909/dataset/40) and a user guide (DOI: 10.16909/dataset/39) to conduct a fast-track Delphi process, upon request to the corresponding author of this publication. Thematic results of the pilot phase are available (in French) in reference [10]. The Supplemental material including supplement to the text body and supplementary Table S1 is made available with this publication.

## Abbreviations

IQR: Interquartile range
NGT: Nominal group technique
RAM: RAND/UCLA appropriateness model
