# Supplementary material for: A multi-stage approach to support timely health policy decisions during crisis: the fast-track Delphi
Source: BMC Public Health. 2024 Dec 18;24:3412. doi: 10.1186/s12889-024-20903-0 (PMC11653821; doi:10.1186/s12889-024-20903-0)
Supplement: Supplementary file 1 — Supplementary Material 1. [file 12889_2024_20903_MOESM1_ESM.docx]

**Supplemental material to: A *fast-track Delphi* approach to support timely health policy decisions during crisis**

**Supplement to the text body**

Based on the methodological procedures and methodological experts’ advice, we developed the *fast-track Delphi* process, which allows for the development and quantification of consensual agreements between thematic experts within two to three weeks. This approach is a hybrid process between a conventional Delphi and a modified version of the Nominal Group Technique (NGT).[^21^](#_ENREF_21) Figure 2 provides an overview of the process, with each step described in the main text body. This supplement to the text body provides more (technical) details on each step, the toolkit development, as well as the results of the feasibility test and evaluations from thematic experts’ feedback.

**Detailed steps of the *fast-track Delphi* process**

*Expert recruitment and identification of target question(s) – Figure 2, panel (a)*

In addition to expert recruitment and target question(s) formulation, the team also identifies one to three pieces of up-to-date literature on the topic (if applicable). The organizational team sends the target question(s) and reference documents to the participating experts along with confirmation of the logistical details for the meeting in Step 1.

*Step 1: an adapted Nominal Group Technique (NGT) – Figure 2, panel (b)*

The organizational team conducts this step according to an adapted NGT, which is qualitative in nature. The expert panel meets in person (recommended modality) and/or virtually. At least one facilitator from the organizational team – ideally two, one of whom manages the *fast-track Delphi* process and the other of whom is an expert on the topic – moderates the meeting. Thematic proposals, i.e. ideas and recommendations from experts in response to the target question(s), are collected, clarified, classified, and selected throughout four phases (see Figure 3):[^6^](#_ENREF_6)^,^[^13^](#_ENREF_13)

1. silent generation (5-10 minutes): each expert privately reflects on the target question(s) and writes down his/her own ideas;
2. round robin (20 minutes): each expert speaks in turn and gives one of his/her ideas generated in (1) until there are no more new ideas to be expressed. One facilitator lists these ideas in real-time on a visible (projected) screen;
3. clarification and categorization discussion (45 minutes): facilitators lead a discussion among experts to clarify their ideas and categorize them (if applicable). This progressive clarification and categorization is consigned in real-time on the visible (projected screen); and
4. prioritization voting (10 minutes): each expert anonymously votes on a subset (ideally 10-15) of these thematic proposals to be addressed in subsequent steps of the *fast-track Delphi* process.

*Step 2: e-questionnaire – Figure 2, panels (c-e)*

This step consists in creating and using an e-questionnaire aiming at collecting experts' opinion on the thematic proposals generated and selected in step 1. The organizational team regroups the list of 10 to 15 thematic proposals considered as priority, i.e., selected during the prioritization vote in step 1, and formulates statements. These statements constitute the e-questionnaire. They allow opinion data to be collected either in the form of ratings or in the form of a response option selection (types of questions detailed in the main text body) and allow experts to request changes to statements by means of free-text comments. The organizational team sends the e-questionnaire to all thematic experts, who complete it within four days.

The organizational team then processes the data and creates a graphical and numerical summary to describe the group results for each statement in a general report, along with comments (qualitative data). Organizers also produce an individualized report for each expert, superimposing his/her opinion on that of the group.

*Step 3: e-questionnaire and result synthesis – Figure 2, panels (f-g)*

The organizational team analyzes the data from step 2 with respects to the definition of consensual (dis)agreement detailed in the main text body, and reformulates, splits, and/or merges statements from step 2 that do not meet the thresholds into new statements. These new statements (ideally all formulated as *rating* questions) form the content of the step 3 e-questionnaire. The organizational team sends this e-questionnaire in a personalized email to all respondents of step 2, along with the individualized report of step 2. Experts complete the e-questionnaire within four days. This re-quantification of their opinion is done considering results of step 2 to encourage the development of consensual agreements.

The organizational team processes the data and combines them with the statements that reached consensual agreement in the previous step to produce a final report. This report includes a summary of the context and goal, methods, and process flow, detailed graphical and numerical descriptions of group results for each statement, and key messages addressed to policymakers.

**Toolkit development**

We identified three key steps that required technical support, namely (1) facilitating the meeting in step 1; (2) administering e-questionnaires in steps 2 and 3; and (3) processing, analyzing, and reporting the qualitative and quantitative data from e-questionnaires at the end of steps 2 and 3. We used or developed the following procedures and tools to handle these key steps.

*MindManager and Mentimeter(-like)*

We strongly recommend a mind map management software for the real-time projected notetaking of the meeting in step 1 (e.g., MindManager software, version 20.0.334). The software should allow to jot down the most important ideas (useful during the round robin) quickly and intuitively, then to reformulate and categorize them while keeping an overview on other proposals (useful during the clarification and categorization phase). In addition, we recommend an online application (e.g. Mentimeter) that allows for easy and anonymous voting for the final phase of step 1.[^4^](#_ENREF_4)^,^[^13^](#_ENREF_13)

*REDCap*

The online administration of step 2 and 3 e-questionnaires requires an electronic survey management platform that guaranties confidentiality and accountability such as REDCap.[^23^](#_ENREF_23)^,^[^24^](#_ENREF_24) We built a generic structure for the three types of statements (rating statement, single option question and multiple-choice question). We built this structure ahead of conducting the process itself, so it could serve as a template during rush periods, i.e., only having to complete it with statement texts to create the e-questionnaire.

*Rstudio*

We strongly recommend an automatized procedure to process data and create generic and individualized reports. We developed a tool coded in R language, using the RStudio interface,[^25^](#_ENREF_25)^,^[^26^](#_ENREF_26) which (1) extracts raw data from the corresponding REDCap project into *.RData databases; (2) processes these databases to correct any labeling mistake and de-duplicate records (in case experts completed the e-questionnaire in several essays separated in time); (3) creates result tables and graphical displays for every statement; and (4) combines these tables and displays with prewritten introductive texts to create generic (group results) and individualized (each expert’s own response with regards to group results) reports. This R code has been tested and revised to run on Microsoft and Mac devices. The code, the associated user guide, and a demonstration code and anonymized dataset will be available upon request to the corresponding author.

**Pilot phase – Feasibility testing**

A total of 38 Swiss French-speaking thematic experts were identified through (1) participants’ list of the Delphi survey conducted by Berlin and colleagues[^28^](#_ENREF_28) and (2) professional contacts established by our team at Unisanté. A subgroup of 13 experts (34%) took part in step 1 (two by videoconference). Ten additional experts took part in the process starting from step 2 (n = 23, 61% of invited experts). Among those 23 respondents of step 2, 21 (91%) also took part in step 3.

Figure 4 shows the flow of statements throughout the *fast-track Delphi* feasibility test. The step 1 meeting took place on June 17, 2022. Two people (one *fast-track Delphi* process expert and one tobacco control expert) co-moderated the meeting. Experts generated 28 thematic proposals and selected 17 of them as priority (four *ex-aequo* items in voting positions 14-17).

The e-questionnaire for step 2 consisted of 21 statements divided into four thematic sections, arbitrarily chosen based on the addressed subtopic. Ten statements (48%) were type 1 statements, 7 (33%) were type 2, and 4 (19%) were type 3 statements. Experts completed the e-questionnaire between June 21 and June 24, 2022. Generic and individualized reports were generated on June 27, 2022. Three statements (14%) reached the required levels of consensual agreement, without comments arguing for a rephrasing of the statement. The remaining 18 statements either: (1) did not reach criteria for consensual agreement or disagreement (1 statement; 5%); (2) were accompanied by comments arguing for a rephrasing of the statement (6 statements; 29%); or (3) were type 2 or 3 statements (11 statements; 52%).

The e-questionnaire for step 3 consisted of 22 new statements derived from rephrasing the 18 statements from step 2 mentioned above, to which 1 statement drawn from experts’ comments was added. All 23 statements were type 1 statements. Experts completed the e-questionnaire between June 28 and July 1, 2022, considering their individualized results from step 2. Generic and individualized reports for step 3 were generated on July 4, 2022. Eighteen statements (78%) reached consensual agreement. Adding the 3 consensual agreements reached at the end of step 2, 21 out of 26 statements (80%) overall reached consensual agreement – result synthesis report generated on July 6, 2022. The remaining 5 either did not reach *agreement* (1 statement with median < 7 on the scale from 1 to 9) or did not reach *consensus* (4 statements with IQR > 3).

**Evaluation of the process by participating thematic experts**

An evaluation questionnaire was sent to all 23 respondents of step 2 from the feasibility test. This questionnaire contained 9 to 12 questions targeting: (a) the relevance of reference documents; (b) the modality preference, added value of the adapted NGT, and moderation quality of step 1; (c) the consideration of group results and comments in elaborating steps 2 and 3; (d) the accessibility and ease-of-use of steps 2 and 3 e-questionnaires, and whether they consulted their individualized step 2 report to complete step 3; and (e) their personal experience with time constraints.

Sixteen experts (70%) completed this questionnaire. Results are shown in Table 2. All respondents recommended the sharing of reference documents upstream of step 1, with an emphasis on the necessary neutrality of the scientific content of these documents (or at least balanced between items). They judged the added value of the adapted NGT procedure for step 1, compared with a conventional Delphi procedure, as very high (8·5 ± 1·3 points (median ± IQR) on the continuous visual analog scale (VAS) from 0 to 10), arguing for an increased expert’s involvement, the possibility of a broad overview of the target question, and a gain of time. For step 1 meeting, the on-site modality appears preferential (added value over a videoconference-only modality judged 9·0 ± 2·0 points), with arguments highlighting a higher interactivity between experts and agility of the discussion moderation. Experts judged the quality of the NGT moderation to 9·0 ± 2·0 points. They rated the taking into consideration of previous step results for the elaboration of the next step e-questionnaire as high as 9·0 ± 2·0 points for step 2 and 9·0 ± 1·0 points for step 3 e-questionnaire, respectively. Some comments highlighted a few ambiguities in statements that contained two items on which they should rate their opinion. All respondents indicated having consulted their individualized step 2 result report when completing the step 3 e-questionnaire. Experts rated the ease-of-use of e-questionnaires as high as 9·0 ± 1·0 points. Comments regarding the coping with time constraints of this *fast-track* process highlighted their appreciation of close deadlines for self-coherence between steps.
