# Supplementary material for: A multi-stage approach to support timely health policy decisions during crisis: the fast-track Delphi
Source: BMC Public Health. 2024 Dec 18;24:3412. doi: 10.1186/s12889-024-20903-0 (PMC11653821; doi:10.1186/s12889-024-20903-0)
Supplement: Supplementary file 2 — Supplementary Material 2. [file 12889_2024_20903_MOESM2_ESM.docx]

**Supplementary table** **to: A *fast-track Delphi* approach to support timely health policy decisions during crisis**

**Table S1: methodological procedures based on the literature review**

|  | **Imperative** | **‘Nice to have’** |
| --- | --- | --- |
| **Identification of target question(s) and expert recruitment**  *Clearly identify and formulate the question you want to address in support to political decision-making, and recruit a balanced and expert panel of participants.* | | |
|  | - Formulate one (or two) target question(s) as broad open-ended. It might originate from scientists themselves, but also from politics and/or other populations concerned by the topic. This question should allow the panel to generate several statements to cover all aspects of the problematic, yet be sufficiently narrow and clearly formulated to avoid any misunderstanding or outside-topic responses.[^5^](#_ENREF_5)^,^[^11^](#_ENREF_11) - Aim to include at least 20 to 25 experts. Based on a predicted 30% refusal at recruitment and dropout rate throughout the process, this ensures a final number of 15 participants.[^3^](#_ENREF_3)^,^[^11^](#_ENREF_11)^,^[^13^](#_ENREF_13)^,^[^18^](#_ENREF_18)^,^[^20^](#_ENREF_20)^,^[^22^](#_ENREF_22) - Select expert participants based on three criteria: - Their current professional experience is in direct link with the target topic; - They confirm their will to revise at each round their initial or previous judgments for the purpose of reaching a consensus; - They are ready to dedicate enough time to answer iterative questionnaires as a priority activity, and whenever possible to take part in the step 1 meeting (date, time and venue pre-defined by the organizational team).[^7^](#_ENREF_7)^,^[^15^](#_ENREF_15)^,^[^17^](#_ENREF_17) - Make sure to include representatives of human social sciences and natural sciences in the panel of experts, as their current professional activities.[^29^](#_ENREF_29) - Do not recruit political or other (governmental) stakeholders who will use the results of the *fast-track Delphi* process for their decision-making.[^22^](#_ENREF_22) - Use personal interaction to recruit participants (in person, by one-to-one phone call or videoconference or via a personalized email), in an effort to maintain a high response rate throughout the process. | - Start by recruiting members of a pre-existing topic-related task force (non-decisional scientific council), i.e., experts already identified at the adequate political and/or geographical level.   When no task force pre-exists or less than 20 participants from the task force agree to participate, one could contact people external to this task force until at least 20 participants meeting all inclusion criteria are included in the panel.   - Such participants are to be identified in priority based on their membership in local or national specialist associations and specific groups, or identified as being study investigators and key authors of studies and publications concerned with the given thematic.[^6^](#_ENREF_6) - As a last resort strategy, one could consider recruiting contacts of already-included participants. This latter strategy needs to be avoided as much as possible to prevent a group homogenization without intending to, as already-included participants might tend to name acquaintances with who they already agree, share ideas and/or background. - Upon recruitment, their confirmation of presence for step 1 (either in-person or virtually) should be collected, thus acting as a psychological sign-up.[^12^](#_ENREF_12) - If applicable, send a corpus of available literature to participants together with the Nominal Group Technique (NGT) meeting notification and target question(s).[^6^](#_ENREF_6) Whenever possible, these literature pieces should comprise a short summary written by the organizational team in the form of highlights bullet points, together with the average judged quality of the given documentation. Moreover, give the possibility to participants to contribute with additional literature (whenever possible, together with a corresponding summary and their judgement on the quality of the source), to be shared with all participants. |
| **Step 1 as an adapted Nominal Group Technique (NGT)** | | |
| *Conduct the first step as a structured brainstorming session, aiming at generating and prioritizing a list of items on which you will seek experts’ opinion.* | | |
|  | - Replace the conventional step 1 by an adapted version of the Nominal Group Technique (NGT) – a face-to-face structured group meeting of experts that is led by an experienced moderator, with the purpose of collecting a listing of statements to cover all aspects of the target question(s).[^6^](#_ENREF_6)   Step 1 should be an idea generation, classification and selection step, not a step focusing on opinion debate.[^13^](#_ENREF_13)^,^[^14^](#_ENREF_14) Thus, this adapted version of the NGT should enable experts to reflect on their own thematic propositions to answer the target question(s), share these propositions with the group, clarify and regroup them into categories, and select the most priority ones.   - Carefully choose a skilled moderator.[^14^](#_ENREF_14) Indeed, the quasi-anonymity principle cannot be fulfilled with the choice of a face-to-face meeting for step 1, i.e. more vocal and outspoken members may dominate the opinion.[^18^](#_ENREF_18)^,^[^19^](#_ENREF_19) - Display the notes and progressive grouping of thematic propositions in real-time during the NGT meeting, for all participants to see. This is especially important for the clarification and categorization discussion, in order to avoid *quiproquos* and ensure an informed vote. | - Whenever feasible, prefer an on-site (or at least hybrid) modality over a videoconference-only meeting. - (*) Conduct this step 1 meeting with a sub-group of the panel of experts if needed, as a function of their short-term availability. That is, experts agreeing to participate in the process may do so starting from step 2, in the case where they are not available for the step 1 meeting. - (*) Insist towards experts that they formulate their ideas as much as possible in a ‘statement-format’ during phases 2 and 3 of this adapted NGT, in order to avoid dealing with too vague propositions when creating step 2 e-questionnaire. |
| **Step 2 by e-questionnaire** | | |
| *Collect experts’ opinion on the list of items generated and selected in step 1, using an e-questionnaire.* | | |
|  | - Collect experts’ opinion, using an e-questionnaire, on thematic propositions generated and selected in step 1 both with quantitative and qualitative (free-text comments) formats.[^7^](#_ENREF_7)^,^[^13^](#_ENREF_13)^,^[^17^](#_ENREF_17)^,^[^19^](#_ENREF_19) Quantitative opinion data might be: - A rating-type of data – we advise the use of 9-point Likert scales with clear labeling of both ends and intermediary points of the scale (1 for complete disagreement to 9 for complete agreement);[^7^](#_ENREF_7)^,^[^13^](#_ENREF_13) - A response-selection-type of data – we advise questions with unique or multiple (maximum three) choice answers without ranking. - Always give the possibility to choose a ‘no answer’ option.[^3^](#_ENREF_3) - Create the e-questionnaire so that each question/statement stands for itself (i.e., no sub-questions). - (*) Conduct a thematic- and a phrasing-review of the e-questionnaire by people other than those who created the e-questionnaire, before sending it. - Ask experts to complete the e-questionnaire within three to four days. - Choose a non-parametrical approach (median ± interquartile range (IQR)) over a parametrical approach (mean ± standard deviation (SD)) for the statistical grouping and description of quantitative data, as it is much more robust to outliers, i.e. extreme ratings.[^3^](#_ENREF_3) Additionally, display results in a graphical format, notably to identify the modality of response distribution.[^3^](#_ENREF_3)^,^[^19^](#_ENREF_19) - Pay attention to assigning detailed and accurate definitions distinguishing the terms *agreement* and *consensus*: the *agreement* for a given statement, i.e., the level of experts’ agreement with the statement itself, is not necessarily equivalent to the *consensus* around a given statement, i.e., the level of experts’ agreement with their peers.[^4^](#_ENREF_4)^,^[^5^](#_ENREF_5)^,^[^12^](#_ENREF_12) The objective of a *fast-track Delphi* process should be to reach *consensual agreement*, i.e., we recommend a definition that takes into account both aspects: - An *agreement* should be considered as reached when the median score is equal or above 7 on a 9-point Likert scale (rating-type of data) or when the response option is selected by at least two third of respondents (response-selection-type of data). When applicable, a disagreement should be considered as reached when the median score is equal or below 3 on a 9-point Likert scale (rating-type of data). - A *consensus* should be considered as reached when the response distribution is unimodal with an interquartile interval (IQR)’s width equal or below 3 points of the 9-point Likert scale (rating-type of data).[^8^](#_ENREF_8)^,^[^12^](#_ENREF_12)   One should remember that the choice of threshold values can only be arbitrary. Therefore, caution should be exercised when interpreting results for those statements whose median and/or IQR fall very close to the thresholds.[^8^](#_ENREF_8) | - Collect data aiming to describe the panel of experts (gender, current position, geographical location whenever applicable). - (*) Collect data describing the current position and/or education background of experts, to be taken into account in result interpretation. Indeed, these parameters might influence the expert’s vision on the topic. - Send an individualized reminder mail or call two days after sending the e-questionnaire, or whenever considered necessary. |
| **Step 3 by e-questionnaire and result synthesis** | | |
| *Use a second e-questionnaire built on step 2 results to push for experts’ consensus development in light of the group’s opinion, and quantify final consensual agreements.* | | |
|  | - Consider only those statements that did not reach consensual agreement by the end of step 2: rephrase, split and/or fuse them according to experts’ responses and comments in step 2 in order to create the e-questionnaire of step 3. An effort should be made so that step 3 e-questionnaire contains as many as possible rating-type of questions (if possible no response-selection-type of questions). - Keep unchanged the exact terms used by experts as much as possible, with modifications of the wording and phrasing kept to what is strictly needed for content aggregation.[^12^](#_ENREF_12) - (*) Conduct a thematic- and a phrasing-reviewing of the e-questionnaire by people other than those who created the e-questionnaire, before sending it. - Inform experts about which statements reached consensual agreement by the end of step 2. - When sending step 3 e-questionnaire, also provide experts with the group and individualized responses from step 2. That is, each expert should receive a graphical and numerical summary of his/her own response in step 2 for each statement, in comparison with the statistically described group response.[^6^](#_ENREF_6)^,^[^7^](#_ENREF_7)^,^[^17^](#_ENREF_17) One should insist towards experts that they are not asked to necessarily change their opinion, but rather to reflect upon a possible change in their opinion in light of the diversity of opinion (and possibly comments) raised by the group.[^3^](#_ENREF_3)^,^[^12^](#_ENREF_12) - Always give the possibility to choose a ‘no answer’ option,[^3^](#_ENREF_3) as well as the opportunity to express any additional comment in a free-text format. - Ask experts to complete the e-questionnaire within three to four days. | - Free-text comments from the preceding round might be provided together with the statistical description of group results (e.g., reference.[^22^](#_ENREF_22)) - Send an individualized reminder mail or call two days after sending the e-questionnaire, or whenever considered necessary. - (*) Depending on the final number of consensual agreements, one may add an extra-step to the process, consisting in a prioritization vote. Experts would be asked to choose the top-3 or top-5 priority consensual statements to be highlighted in the communication to decision-makers. This extra-step might be conducted over 24 hours, and using the same tool and procedure as those used at the end of step 1 (prioritization vote during the adapted NGT). |

(*) denotes extra methodological procedures added after the feasibility test, based either on thematic experts’ feedback or on self-critique. IQR: interquartile range. NGT: nominal group technique. SD: standard deviation.
